# Scent Detection Threshold of Trained Dogs to *Eucalyptus* Hydrolat

**DOI:** 10.3390/ani14071083

**Published:** 2024-04-03

**Authors:** Soile Turunen, Susanna Paavilainen, Jouko Vepsäläinen, Anna Hielm-Björkman

**Affiliations:** 1 School of Pharmacy, Faculty of Health Sciences, University of Eastern Finland, 70211 Kuopio, Finland; soile.turunen@uef.fi (S.T.); jouko.vepsalainen@uef.fi (J.V.); 2 Wise Nose-Finnish Odor Separation Association, 00790 Helsinki, Finland; susanna.paavilainen@noseacademy.fi; 3 Nose Academy Ltd., 70780 Kuopio, Finland; 4 DogRisk Research Group, Department of Equine and Small Animal Medicine, Faculty of Veterinary Medicine, University of Helsinki, 00014 Helsinki, Finland

**Keywords:** canine, olfaction, eucalyptol, hydrolat, nose work, line-up

## Abstract

**Simple Summary:**

Dogs have an extraordinary sense of smell that is far superior to humans’, thanks to their unique anatomy and physiology. This remarkable ability allows them to detect and differentiate between very low concentrations of odor molecules, but the threshold seems to depend on the target odor. This study focused on determining the lowest concentration of *Eucalyptus* hydrolat that would be detectable by trained dogs. This substance was selected for the study as it is used in a dog scent training sport called “nose work”. The research involved testing dogs with progressively diluted concentrations of this hydrolat until they could no longer identify it, thus determining their scent detection threshold. When dogs were trained to respond to the *Eucalyptus* hydrolat at decreasing concentrations, they successfully detected the scent even when it was diluted to ratios between 1:10^17^ and 1:10^21^. The study also used analytical spectroscopy to analyze the contents of ten commercial *Eucalyptus* hydrolats, revealing variations in their ingredients. The findings highlight two key points. First, with appropriate training, dogs can learn to identify very low concentrations of *Eucalyptus* hydrolat. Second, the consistency of the scent source is crucial in training a dog, not only in canine sport competitions, but also in olfactory research.

**Abstract:**

Dogs’ (*Canis lupus familiaris*) sense of smell is based on a unique anatomy and physiology that enables them to find and differentiate low concentrations of odor molecules. This ability is exploited when dogs are trained as search, rescue, or medical detection dogs. We performed a three-part study to explore the scent detection threshold of 15 dogs to an in-house-made *Eucalyptus* hydrolat. Here, decreasing concentrations of the hydrolat were tested using a three-alternative forced-choice method until the first incorrect response, which defined the limit of scent detection for each tested dog. Quantitative proton nuclear magnetic resonance spectroscopy was used to identify and measure the contents of ten commercial *Eucalyptus* hydrolats, which are used in a dog scent training sport called “nose work”. In this study, the dogs’ limit of detection initially ranged from 1:10^4^ to 1:10^23^ but narrowed down to 1:10^17^–1:10^21^ after a training period. The results show that, with training, dogs learn to discriminate decreasing concentrations of a target scent, and that dogs can discriminate *Eucalyptus* hydrolat at very low concentrations. We also detected different concentrations of eucalyptol and lower alcohols in the hydrolat products and highlight the importance of using an identical source of a scent in training a dog for participation in canine scent sport competitions and in olfactory research.

## 1. Introduction

It is well-known that dogs have an excellent sense of smell [1]. The anatomy and physiology of the nasal structures of dogs lend themselves especially well to conveying the airflow to the olfactory epithelium of the nose [2]. The nasal epithelium contains olfactory receptor (OR) cells which possess ORs coded by a wide repertoire of polymorphic OR genes [3]. Compared to humans, dogs have more OR coding genes, but fewer of them are dormant [4]. The nerve signal is triggered by odor molecules after they bind to the specific ORs. The signal projects into the olfactory bulb, which is more predominant in dogs than in humans [5]. Due to these special characteristics, it is possible for dogs to detect odor molecules at very low concentrations.

Conventionally, dogs’ superior ability for odor discrimination is employed for many purposes, e.g., by the police and customs officials, as they train service dogs to detect substances such as narcotics [6], semen stains [7], ignitable liquids [8], explosives [9], etc. Dogs can detect the target scents even in very demanding conditions, such as in varying temperatures and humidities, as well as with other distractors [10,11]. This skill is widely utilized, e.g., when rescue, search, and hunting dogs seek and trace their targets, dead or alive, on/in/under water, soil, or snow. Despite some technical and ethical challenges, there has been considerable interest in medical scent detection dogs regarding SARS-CoV-2 detection [12,13,14,15,16]. The usage of disease scent dogs has been also studied in connection with cancer [17,18,19,20,21], Parkinson’s disease [22], malaria [23], *E-Coli*-bacteriuria [24], and *Clostridium difficile* infection detection [25], among others.

In addition to service and medical scent detection dogs, several other canine scent detection alternatives have become popular. Dogs have been trained to detect many scents, e.g., truffles in the ground [26], mold in houses [27], and bed bugs in apartments and hotels [28]. Both a dog sport and a hobby, nose work was developed in the USA in 2006. In this scent training sport, dogs are trained to detect and indicate the scents of birch, anise, and cloves from various search areas [29]. In Finland, nose work dogs detect three alternative target scents, namely Eucalyptus, bay leaf, and lavender [30]. In nose work sports, the scents are usually presented to the dogs using commercial hydrolats. These are obtained as a by-product during the steam distillation of a target plant when producing its essential oil [31]. These hydrolats are reported to contain water and dissolved natural compounds.

As dogs and their scent-detecting capabilities are widely used, there is an increasing interest to quantify the thresholds of their olfactory sensitivity. Walker et al. used a “find the target” method, where they built an olfactometer to determine accurately the olfactory threshold of two dogs to amyl acetate, a compound which has a banana-like odor, from the air [1,32]. Olfactometers were also used when determining dogs’ threshold to detect the Spotted Lantern fly (a foreign species in the USA) [33], and when evaluating dogs’ generalization and discrimination across isoamyl acetate concentrations [34]. In addition, different kinds of apparatus are nowadays applied in scent detection training and testing that present target odors using scent tracks, scent line-ups, cones, carousels, and other scent wheels. In these, the target odors or samples are placed in open or mesh-covered jars, where the odor molecules are transferred into the air by passive diffusion and with the help of the dog’s sniffing. Concha et al. tested dogs’ olfactory threshold with amyl acetate using an eight-choice carousel [35], whereas DeChant et al. selected isoamyl acetate as a target for a three-alternative forced-choice test in a scent line-up device [36]. Other studies conducted in applied animal science and forensic science have used scent line-ups or carousels when investigating dogs’ olfactory threshold to other targets, e.g., tuatara and gecko scents [37], koi carp [38], or ignitable liquids [8], as well as when studying olfactory properties of dogs and wolves in detecting raw food [39]. Detection thresholds for odors can be determined using different kinds of methods in both humans and dogs [40]. Studies in canine olfactory threshold research exploiting a staircase procedure have recently been reported in conjunction with a step-down procedure [33,36]. Whereas step-down procedures include one or several exposures to each concentration of a target odor before introducing the next lower concentration of the target, the staircase procedure applies pre-defined steps where the concentration of a target odor is either increased or decreased according to the dog’s failure or success to indicate the target, respectively.

The aim of this study was to determine the scent detection threshold of trained dogs to an in-house-made *Eucalyptus* hydrolat, a source of an unambiguous scent, eucalyptol. The dog’s threshold for the target scent was determined by using a three-alternative forced-choice procedure applying scent line-ups. Each target sample was presented in a descending concentration order. A total of 15 dogs were tested in double-blinded, randomized sample settings where the last correct indication was recorded as the individual threshold.

## 2. Materials and Methods

### 2.1. Preparation of In-House Eucalyptus Hydrolat

A pure (100%) essential oil of *Eucalyptus radiata* (Frantsila, Kyröskoski, Finland) was purchased as the source of the target scent. To prepare the stock solution (i.e., in-house *Eucalyptus* hydrolat) the *Eucalyptus* oil was diluted with class 1 ultra-pure water (Elga Purelab Ultra Analytical, High Wycombe, UK). The stock solution contained the ratio of 1:10,000 (1:10^4^) oil in water (0.1 mL:999.9 mL), mimicking the average concentration of the *Eucalyptus* hydrolats used in dog nose work sports. The stock solutions were prepared in a laboratory in the School of Pharmacy of the University of Eastern Finland with clean laboratory glassware into a one-liter volumetric flask and transported to the test site at room temperature. The above-mentioned class 1 ultra-pure water was used for further dilutions and for non-target samples.

### 2.2. Preparation of In-House Eucalyptus Hydrolat Dilutions

The stock solution was diluted in the test sites to prepare the odor samples as follows: Researcher 1 was responsible for diluting the solutions as described in Table 1 and Supplementary Tables S1 and S2 in a space separate from the testing room. Every diluted solution utilized a glass graduated pipette to exactly measure 0.1 mL or 1.0 mL of the previous diluted solution into a 20 mL transparent glass bottle, in which 9.9 mL or 9.0 mL of class 1 ultra-pure water was added, respectively. The bottle was capped, inverted, and shaken to obtain a homogenous solution. For the non-target samples, parallel bottles were filled with 10.0 mL of the class 1 ultra-pure water. Disposable powder-free nitrile gloves were used when preparing the dilutions. A new pair of gloves was changed when starting to prepare the next solution.

### 2.3. Preparation of Target and Non-Target Samples

A vortex mixer was used to mix the prepared-in-house *Eucalyptus* hydrolat dilutions. A one milliliter (1.0 mL) sample of the diluted solution, i.e., the target sample, as well as 1.0 mL of the class 1 pure water, i.e., the non-target sample, were transferred from their glass bottles into single-use PP-plastic cups (ABENA drinking cup, 200 mL, white PP, Denmark) using their designated graduated pipettes. Researcher 1 transferred the target and non-target samples into the plastic cups immediately before testing each scent line-up to ensure uniformity of sample processing for each tested dog.

### 2.4. Scent Line-Up Tracks for a Three-Alternative Forced-Choice Method

**Figure 1:**
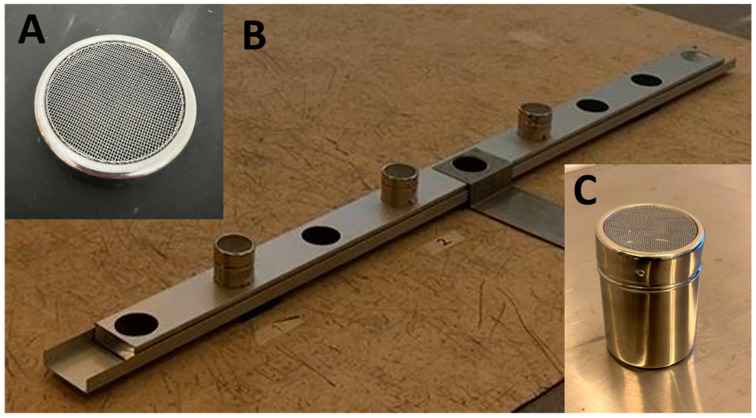
A metallic scent track was used as an apparatus for a three-alternative forced-choice task in Studies 1 and 2 (**A**); mesh cover (**B**); scent track with three metallic jars placed on every second place from the left (**C**); mesh-covered metallic jar containing the plastic cup with target or non-target sample.

**Figure 2:**
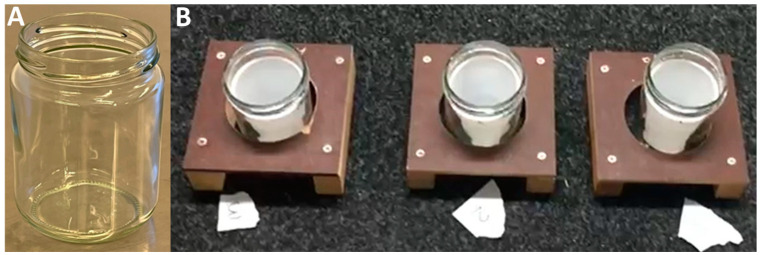
Individual plywood stands were used in Study 3 as a three-alternative forced-choice device (**A**); glass jar (**B**); glass jars containing the plastic cup with target or non-target sample.

Researcher 2 was responsible for placing the samples prepared by Researcher 1 as follows: one target and two non-target samples were placed into clean mesh-covered metallic jars (stainless-steel, 85 mm high and 70 mm in diameter, Figure 1) or clean glass jars (clear glass, 98 mm high and 67 mm in diameter, Figure 2). The jars were transferred to the test room and placed in the scent track according to a computer-generated randomization list (www.sealedenvelope.com, accessed on 31 May 2017). A total of three jars were placed in a metallic scent track with nine holes for the metallic jars (in Study 1 and 2, Figure 1), or in three individual plywood stands holding the glass jars (in Study 3, Figure 2). These three jars constituted a scent track for line-up odor discrimination using a three-alternative forced-choice method. Disposable powder-free nitrile gloves were used and changed between every tested scent line-up. Mesh covers and metallic and glass jars were used only once, i.e., for one sample, during each test day.

### 2.5. Test Protocol

The dilutions were presented in descending order starting from the strongest concentration (stock solution, 1:10,000). The indication was recorded as correct if the dog alerted a jar with the target sample, using its individual-specific alerting behavior (focused stare, nose freeze, sitting or lying down in front of the indicated jar, or pawing the jar) for a minimum of two seconds. The indication was recorded as incorrect if the dog indicated a jar with the non-target sample by alerting as mentioned above. A lack of indication was recorded if the dog did not indicate any of the three samples, but simply left all three jars without making any alerting behavior. Distractor scents were not used because the study aim was not to test the specificity of the dogs towards *Eucalyptus* hydrolat, but to determine the threshold of the lowest ratio in aqueous solution that individual dogs could discriminate from pure water.

One dog at a time performed the discrimination test in a double-blind manner, i.e., neither the dog nor the handler knew the status of the samples in the scent line-up. Handlers were instructed to give their dogs a signal to start the discrimination test and then to report the dog’s target indication (sample number 1, 2, or 3) out loud. The handler received feedback of the result (vocally YES/NO) and rewarded the dog with verbal praise or a clicker paired with treats for the correct indication.

Two people kept notes of the handlers’ reporting and one external assessor observed the tests in the test room. Only the external assessor was blinded to the sample status. The test results were interpreted as follows: All consecutive indications had to be correct. An incorrect indication or lack of indication led to a termination of the test. As the threshold value for each tested dog, the lowest correctly indicated concentration of diluted in-house-made *Eucalyptus* hydrolat was reported as a value of the volume fraction, i.e., ratio of *Eucalyptus* oil to water (mL:mL).

### 2.6. Study 1

This study aimed to determine the scent detection threshold of nose work sport dogs to an in-house-made *Eucalyptus* hydrolat. Eleven dogs training nose work sports, and one dog with no sporting experience of nose work but an ability to identify several other target scents, were recruited for Study 1 with their handlers. Details of the dogs are presented in Table 2. All recruited nose work sports dogs were either hobbyists or contestants of the sport in Finland. They had been trained with different commercial *Eucalyptus* hydrolats, which are used as sources of the scent of Eucalyptus by the Finnish Nose Work Club. As none of these dogs had encountered the produced-in-house *Eucalyptus* hydrolat before the test day, their handlers were allowed to familiarize the dog with the target scent as follows: The dogs were allowed to sniff one mL of the stock solution from a jar, followed by a clicker sound and a treat for positive reinforcement training of the target sample, for a maximum of five times. Handlers were solely responsible for deciding whether to conduct the familiarization procedure or not. The test started a few minutes after the familiarization process had been completed.

In the test, handlers were allowed to estimate and determine the strength of the correct indication. If the dog’s indication was correct and stated as strong, the test proceeded. If the indication was stated as weak, the dog was allowed to search the same samples again in a new order up to four times. The jars were re-arranged in the line-up by Researcher 2 out of sight of the dog handler pair. This seemingly subjective decision was not used as an assessment method, as the first incorrect or lack of indication ended the test for the dog. Instead, repetition was used as a means of reinforcing the alerting behavior to the target sample after a weak indication.

One dog with no prior experience of nose work sports (Dog 1) had a brief training session before the trial. First, Dog 1 was allowed to sniff 1 mL of stock solution from a jar, followed by a clicker sound and a treat for positive reinforcement training of the target sample, for a total five times. Second, five training scent line-ups were performed mimicking the test protocol, and again, Dog 1 was rewarded with a clicker and treats for the correct Indication of stock solution. Study 1 was conducted at the Wise Nose dog training center in Viikki, Finland in June 2017.

### 2.7. Study 2

Study 2 was conducted to re-test and verify Dog 1’s surprisingly low threshold to the in-house *Eucalyptus* hydrolat recorded in Study 1. The same test protocol was applied as described in Study 1. Here, Dog 1 was again allowed to sniff a 1 mL sample of the stock solution from a jar, followed by a clicker sound and a treat for positive reinforcement training of the target sample, for a total five times before starting the test. Study 2 was conducted at the Wise Nose dog training center in Viikki, Finland in June 2017.

### 2.8. Study 3

Based on the results from Studies 1 and 2, the aim of this study was first to train the dogs to discriminate the in-house *Eucalyptus* hydrolat in decreasing concentrations from the pure water and then to conduct the testing. Dog 1 was included to test his scent detection threshold in another location under different circumstances and with a different apparatus compared to Studies 1 and 2. Three new dog handler pairs were recruited for Study 3 (Table 2), with the dogs being trained by their handlers in the Vainuvoima scent training center a total of 14 times during a four-month period. In the training, the stock solution was diluted as described in the study protocol and in Supplementary Table S2. One training session comprised of an average of 20 scent line-ups per dog where one line-up contained 0 or 1 target sample of diluted solution and 3 to 5 non-target samples. Several different concentrations were used in one training session. Their training was based on the positive reinforcement method, i.e., the dogs were rewarded with a clicker and treats when they correctly indicated the diluted solutions. The dogs were tested similarly as described in Study 1. For these dogs, no prior training occurred on the test day of Study 3. Dog 1 was again allowed to sniff a 1 mL sample of the stock solution from a jar, followed by a clicker sound and a treat for positive reinforcement training of the target, for a total of five times. The study was conducted in the Vainuvoima scent training center in Loimaa, Finland in April 2018.

### 2.9. Ethics Statement

This research does not include the kind of experimental setup that would demand applying for an ethical statement from the ethics committee, according to Finnish research ethics. Voluntary participants were recruited for tests conducted with a harmless scent product and no data was collected from dog owners and/or dog handlers.

### 2.10. NMR Analysis of the Eucalyptus Oil and Hydrolats

Essential *Eucalyptus* oil, as well as ten different *Eucalyptus* hydrolat solutions used in nose work sports training and competitions, were purchased from online stores. The content of the oil and hydrolats were measured using the nuclear magnetic resonance (NMR) technique as follows: ^1^H NMR spectra were measured using a 600 MHz Bruker NMR spectrometer equipped with a cryoprobe (Bruker Prodigy TCI 600 S3 H&F-C/N-D-05 Z) and an automatic SampleJet sample changer. Prior to the NMR measurements, 100 µL of a sample was transferred to a 5 mm NMR tube, followed by D_2_O (425 µL) containing 1.0 mM 3 (trimethylsilyl)-propionic-d4 acid (TSP) as an internal standard of known concentration. The *Eucalyptus* oil was measured as CD_3_OD. Compounds were identified by using separately measured reference compounds. ^1^H NMR spectra were collected using the zg automation program with the following parameters: 90° pulse angle, total relaxation delay 13 s, and 32 scans at 300 K. The concentrations of the identified compounds (X) from the NMR spectrum are calculated using the equation:

c(X) = (A(X)/A(Y) × N(Y)/N(X) × c(Y))

where N is the number of protons producing the signal and A is the area of the signal. Y is the reference compound with the known concentration, for example, TSP. Typically, ^1^H NMR detection limits depend on measuring time and the compound’s structure. In this study, a measuring time of approximately 6 min with 32 scans was used, making it possible to detect 0.002 millimolar and to quantify 0.01 millimolar (i.e., 1 mg/L) concentrations of small molecules (molecular weight < 500 g/mol).

## 3. Results

### 3.1. Scent Detection Threshold

**Figure 3:**
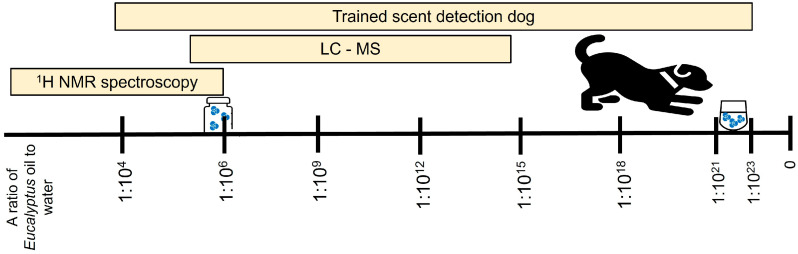
The olfactory threshold of the trained dogs outperforms typical analytical techniques. While trained sniffer dogs needed a minimum of only 1:10^21^–1:10^23^ of *Eucalyptus* oil in an aqueous solution for odor indication, the detection limit of ^1^H NMR spectroscopy was 2:10^6^ in this study. For comparison, it should be noted that basic mass spectrometry methods coupled with liquid chromatography detect common chemical compounds in liquids at 1:10^15^ levels, but this technique was not utilized here. The figure is not scaled. ^1^H NMR, proton nuclear magnetic resonance; LC-MS, liquid chromatography-mass spectrometry.

In Study 1, two of the 11 nose work sports dogs indicated the diluted-in-house *Eucalyptus* hydrolat at a ratio of 1:10^10^, whereas three of the dogs only indicated the stock solution at the highest concentration of 1:10^4^ (Table 2). The rest of the nose work sports dogs indicated other concentrations in between these levels. Dog 1 was the only animal that indicated all dilutions between the dilution ratios of 1:10^4^ and 1:10^22^. In the second study (2), all tested dilution ratios from 1:10^4^ to 1:10^23^ were indicated by Dog 1. In the third study (3), two dogs indicated samples up to a dilution ratio of 1:10^21^ (Dogs 1 and 15), one to a ratio of 1:10^19^ (Dog 13) and one to a ratio of 1:10^17^ (Dog 14). The results are presented in Table 2. The scent detection thresholds of trained dogs to *Eucalyptus* hydrolat are illustrated in Figure 3.

### 3.2. NMR Detection and Identification of Compounds

**Figure 4:**
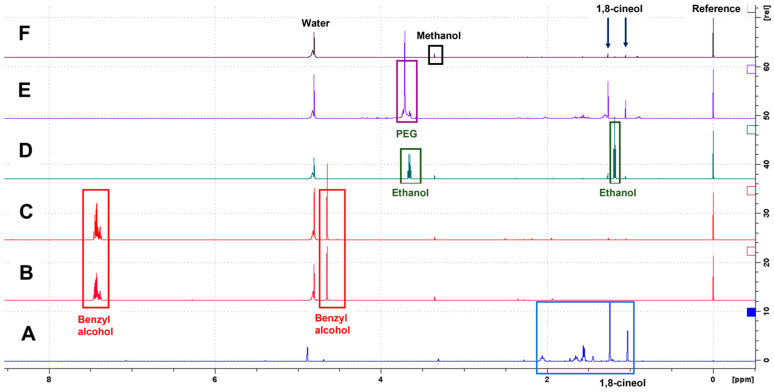
^1^H NMR spectra revealed the existence of different ingredients in the *Eucalyptus* hydrolats. (**A**) The spectrum shows the typical signals of eucalyptol (blue box) in the sample of the 100% essential oil of *Eucalyptus radiata* (Frantsila, Kyröskoski, Finland). The quantified concentration of eucalyptol was 4.12 mol/L. (**B**–**F**) The spectra show signals from benzyl alcohol (red boxes), ethanol (green boxes), polyethylene glycol (PEG, purple box), and methanol (black box) in different *Eucalyptus* hydrolats. In addition, signals of water and the standard compound TSP (Reference) are named, and the two characteristic signals of eucalyptol (1,8-cineol) are highlighted with arrows.

When the content of the essential *Eucalyptus* oil and the ten purchased *Eucalyptus* hydrolats were measured using NMR spectroscopy, the analysis revealed a large variation in the concentration of eucalyptol between the different products. The oil contained 4.12 mol/L of eucalyptol, which was identified as the main compound of the oil (Figure 4A). On the contrary, some of the hydrolats contained less eucalyptol than the limit of detection (C(eucalyptol) min. < 0.01 mmol/L, max. 5.05 mmol/L) (Figure 4B–F). In addition, three alcohols with varying concentrations were identified in the hydrolats: ethanol (min. < 0.01 mmol/L, max. 32.72 mmol/L), methanol (min. < 0.01 mmol/L, max. 1.15 mmol/L), and benzyl alcohol (min. < 0.01 mmol/L, max. 42.68 mmol/L). Some of the identified ingredients included acetic acid and formic acid (for detailed information, see Supplementary Table S3). Examples of the NMR spectra of different *Eucalyptus* hydrolats and the *Eucalyptus* oil used in this study are presented in Figure 4 and Supplementary Figure S1.

## 4. Discussion

The results of this study indicate that it is possible to train dogs with decreasing concentrations of a target scent to improve their olfactory threshold. Here, eleven nose work sport dogs were tested using an in-house-prepared *Eucalyptus* hydrolat in Study 1. Though the dogs had previously been training for nose work with commercial *Eucalyptus* hydrolat products and some of them were even competing in nose work sports, these dogs were able to indicate only substantially higher concentrations of the in-house *Eucalyptus* hydrolat in comparison to the dogs in Study 3. In contrast to Study 1, the three dogs in Study 3 were trained with diluted target scent solutions, and when tested, all of them successfully indicated very low concentrations of the in-house *Eucalyptus* hydrolat.

The difference between the results of Study 1 and Study 3 can partially be explained by the prior training with the descending concentrations of the target scent in Study 3. DeChant et al. reported that training at lower concentrations of the target odor improved the dogs’ threshold to isoamyl acetate as compared to dogs that were trained with only one concentration [36]. Our findings are in agreement with DeChant et al., i.e., that training with ever-decreasing concentrations of a target scent might have enhanced the dogs’ generalization to the *Eucalyptus* hydrolat, possibly by changing the physiology of the olfactory system. Rodent studies have suggested that training may be able to increase the number of OR cells specific to a target scent molecule and increase the sensitivity towards the molecule [41,42], but for dogs, this remains a research question for the future. The second methodological aspect is that we conducted this study utilizing the three-alternative forced-choice scent line-up method, using scent tracks and jars for samples. The scent line-up method has been used in several studies, e.g., to test dogs’ olfactory threshold to isoamyl acetate [36] and ignitable liquid [8], as well as in SARS-CoV-19 detection [15]. However, some of the nose work sport dogs in Study 1 were not familiar with this method. Instead, they were accustomed to finding hidden target scents in various environments which greatly differ from working with a scent line-up. This was overcome in Study 3, where the scent line-up method was applied already in the training period, ensuring that the dogs were prepared for the test.

The third technical consideration emerges from the NMR measurements of the ten commercial *Eucalyptus* hydrolat products. The NMR spectra revealed the presence of several substances in the purchased *Eucalyptus* hydrolats, e.g., the presence of lower alcohols such as ethanol and benzyl alcohol, which were not detected in the essential *Eucalyptus* oil. In addition, the concentrations of these lower alcohols varied substantially between the products. According to the literature, a steam distillation process does not involve lower alcohols. Instead, ethanol and propylene glycol are used to remove unstable terpenes from essential oils to obtain more flavor and increase the product’s shelf life [43]. In addition to these alcohols, the concentration of eucalyptol, the compound with a characteristic smell of eucalyptus, differed between the analyzed products by up to more than 500-fold. Three out of the ten hydrolat products contained less than the quantification limit, 0.01 mmol/L of eucalyptol, according to the NMR analysis. This is evidence that the hydrolats used in nose work sport training and competitions are not standardized. These hydrolats also differed from the target scent used in Study 1. In fact, hydrolat products from different manufacturers might lead to totally different odor patterns for dogs due to their different amounts of volatile organic compounds (VOCs). The VOCs are affected by the ingredients and their concentrations. As a possible consequence, problems may arise in nose work sporting competitions when a dog searches for a scent of *Eucalyptus* hydrolat that differs from the one(s) with which he/she has been trained. In the case of nose work, a sport in which national and international competitions are held, it would be extremely important for the scent products to be standardized, both in terms of ingredients and concentrations.

Additionally, one dog (Dog 1) was tested three times within this project. He had been trained to discriminate between several different target odors using the scent line-up method before participating in the studies. However, this 5-year-old neutered male dog had no prior training with any *Eucalyptus* hydrolats (i.e., no experience of nose work sports), but the handler trained him quickly to detect the target scent before testing him in each of the three trials. In Study 1, he already achieved an astonishing threshold of 1:10^22^. When he was re-tested in Study 2, he could discriminate the in-house-made *Eucalyptus* hydrolat at the lowest ratio recorded in this study (1:10^23^). In Study 3, he reached the ratio of 1:10^21^, showing reproducible results. We assume that this dog was successful because he had a very highly developed sense of smell, he was quick at learning new targets, and he had already mastered several different odor targets, including early-stage cancers. In fact, research has shown that dogs with more trained targets tend to learn a new one more easily [44,45], and only 2 to 3 exposures are needed for such dogs to learn to respond to a novel target odor [46]. Individual differences between the scent detection dogs, as seen here in our study, have been observed in most studies. Several reasons have been proposed to explain this phenomenon, including the personality of the dog and its training and motivation [35]. In addition, there are differences in the anatomy and physiology of the olfactory organs of different breeds of dogs. Dogs have been bred from different genetic backgrounds, leading to different sizes of the olfactory epithelium, as well as the number and type of ORs present in an individual dog’s epithelium [47,48]. The large OR gene repertoire (1094 genes for dogs) and the high level of polymorphism in canine OR genes have been discussed alongside signal transduction and brain processing when trying to find an explanation for the extremely sensitized sense of smell of these animals [47].

There are several limitations to this study. The criterion of “first incorrect response” was chosen as indicating the dogs’ threshold for *Eucalyptus* hydrolat, which did not allow us to statistically determine the true and absolute threshold. As dogs make errors for a variety of behavioral and situational reasons by performing a repetition of every concentration, it would have allowed a statistical estimate of threshold variability for each individual dog. Thresholds for odors can be studied using different kinds of methods in both humans and dogs: both the step-down and the staircase method usually involve several exposures to each concentration, allowing for calculations of performance metrics [40]. In addition, we chose a three-alternative forced-choice procedure, whereas at least four or five alternatives would have achieved a more satisfactory measurement of the threshold. Wise et al. described the three-alternative forced-choice method with ascending concentrations (FC-AML) where the individual concentration was presented only once, but this made it impossible to calculate estimates of correct proportions for any given concentration [40]. However, FC-AML has been suggested to be a valid tool for characterizing both average threshold and individual differences. We applied these FC-AML method criteria in a post hoc analysis for our results as follows: Firstly, the dog responded correctly in the last three consecutive trials with the descending concentration of the target scent; secondly, the handler was sure that the dog’s last indication was correct. A total of nine out of 15 tested dogs met these criteria when the dilution ratio of 1:10^8^ was the target in the third trial, whereas all dogs in Study 3 were able to indicate the target odor in a minimum of seven and a maximum of nine consecutive trials where the probability of correctly indicating the consecutive tracks was 0.333^7^ (*p* = 0.00045) for seven trials and 0.333^9^ (*p* = 0.00005) for nine trials. However, these values should be interpreted with caution because no power analysis was made to determine the effect size. In addition, it is essential to consider the context as well as potential biases when interpreting results with a *p*-value less than 0.05.

The usage of a liquid dilution in threshold testing might produce inconsistency between the solutions compared to air dilution [36]. Here, we used the same dilution method across all three studies and transferred the target and non-target samples into plastic cups and jars just before presenting them to a dog. We estimated that this procedure made no temporal difference in how the target odor evaporated and equilibrated in the jar between the trials for a dog, but that it harmonized the study set-up between the dogs. In addition, the volatile compounds from a liquid sample would be transferred into the air by passive diffusion. The active sniffing by the dog causes the odor molecules to gain access to the dog’s nasal olfactory epithelium [10]. Thus, the significance of using a liquid dilution is considered to be minor. Moreover, the testing procedures were performed indoors during June 2017 (Studies 1 and 2 were in the Wise Nose training center) and April 2018 (Study 3 was in the Vainuvoima training center). The humidity and the temperature of the room were not recorded, except for Study 2 (humidity 39% and temperature 22 °C). We presume that for Study 1 and 3 the values would have been similar to those present in Study 2, but we do acknowledge that the humidity and temperature should be recorded when studying scent detection capabilities of dogs.

The final limitation was that the essential *Eucalyptus* oil used in these three studies was not identical to the commercial *Eucalyptus* hydrolat products that were analyzed in this study. The essential *Eucalyptus* oil is a mix of several substances. Studies conducted with gas chromatography-mass spectrometry (GC-MS) have identified VOCs, e.g., monoterpene hydrocarbons such as limonene, and other oxygenated monoterpenes such as α-terpineol in *Eucalyptus* oils [49,50]. In general, the same VOCs are also found in *Eucalyptus* hydrolats. These are natural compounds originating from Eucalyptus tree leaves and stems, and they are likely also part of the VOC pattern detected by the dogs. Nevertheless, we chose to use the essential oil, as we hypothesized that its aqueous dilutions would be the best way to simulate the *Eucalyptus* hydrolats. In addition, the concentration of the main component of the *Eucalyptus* oil, eucalyptol, was high enough to conduct a serial dilution; further, eucalyptol is miscible in 3500 mg/L water (at 21 °C) [51]. However, the diluted target solutions were not measured using the NMR spectroscopy technique, as such low concentrations cannot be detected with this kind of analytical technique (see text of Figure 3). In the future, when studying dogs’ threshold to detect the pure odor of eucalyptol, it would be preferable to utilize a commercial product of 99% of eucalyptol (synonym 1,8 cineol) instead of an essential oil. In addition, GC-MS could be applied to investigate the VOC pattern and to quantify the main scent-producing compounds present in the sample down to the detection limit of the analytical technique.

## 5. Conclusions

In conclusion, dogs can be trained to indicate extremely low concentrations of compounds vaporizing from aqueous samples, concentrations clearly below the detection threshold of sophisticated analytical instruments used today, as well as far below what has previously been reported for dogs. The contents of *Eucalyptus* hydrolats are highly variable, which may result in inequality when these are used in nose work sports.

## Figures and Tables

**Table 1:** Prepared dilutions using the in-house-made *Eucalyptus* hydrolat, i.e., stock solution and water, in Study 1. Dilution ratios of *Eucalyptus* oil in water are depicted as the value of a volume fraction.

Solution	Dilution Ration
stock solution	1:10^4^
dilution 1	1:10^6^
dilution 2	1:10^8^
dilution 3	1:10^10^
dilution 4	1:10^12^
dilution 5	1:10^14^
dilution 6	1:10^16^
dilution 7	1:10^18^
dilution 8	1:10^20^
dilution 9	1:10^22^
dilution 10	1:10^24^

**Table 2:** Demographics and results of the dogs in Studies 1–3.

Dog	Breed	Age	Sex	Neutered Status	Trained Target Scents	Level in Nose Work Sports	Last Ratio of *E.* hydrolat Successfully Indicated by Dog		
							**Study 1**	**Study 2**	**Study 3**
1	Spanish Galgo mix	5 y	M	Y	bedbugs, tracking of rats, molds, floor carpet glue residues, cancer	no experience	1:10^22^	1:10^23^	1:10^21^
2	Parson Russell terrier	1 y 9 m	F	N	eucalyptus	hobbyist	1:10^4^		
3	Parson Russell terrier	2 y 7 m	M	Y	eucalyptus, bay leaf, lavender, pieces of Kong toy	contestant	1:10^6^		
4	Beagle	2 y 2 m	M	Y	eucalyptus, bay leaf, lavender, chanterelle, tracking of blood, human, and dog scents	contestant	1:10^6^		
5	Cavalier King Charles spaniel	5 y 2 m	M	N	eucalyptus, bay leaf, lavender	hobbyist	1:10^8^		
6	Giant schnauzer	8 y 5 m	F	Y	eucalyptus, bay leaf, lavender	hobbyist	1:10^6^		
7	French bulldog	2 y 6 m	M	N	eucalyptus, bay leaf	hobbyist	1:10^8^		
8	Mittelspitz	6 y 6 m	F	N	eucalyptus	contestant	1:10^10^		
9	Shepherd mix	4 y 0 m	F	N	eucalyptus, human scent	hobbyist	1:10^10^		
10	Parson Russell terrier	5 y 10 m	F	N	eucalyptus, chanterelle	hobbyist	1:10^4^		
11	Dogo Argentino	5 y 0 m	F	N	ID tracking of human, district heating water, eucalyptus	hobbyist	1:10^4^		
12	Long-haired Dutch shepherd	4 y 2 m	M	NA	eucalyptus	hobbyist	1:10^8^		
13	rough collie	5 y 7 m	M	N	eucalyptus	hobbyist			1:10^19^
14	Danish-Swedish farm dog	5 y 4 m	F	Y	cancer	no experience			1:10^17^
15	Australian kelpie	5 y 7 m	F	Y	eucalyptus, bay leaf, lavender	hobbyist			1:10^21^

y, years; m, months; M, male; F, female; Y, yes (sterilized); N, no (not sterilized); NA, not available; hobbyist, dog has participated in nose work sports exercises but not competed yet; contestant, dog has participated in an official nose work sports competition in Finland.

## Data Availability

The original contributions presented in the study are included in the article and Supplementary Material. Further inquiries can be directed to the corresponding author.

## Supplementary Materials

The following supporting information can be downloaded at: https://www.mdpi.com/article/10.3390/ani14071083/s1, Figure S1: Expansion of methyl region of ^1^H NMR spectra shows the structure of eucalyptol, the main compound in *Eucalyptus* oil; Table S1: Prepared dilutions in Study 2; Table S2: Prepared dilutions in Study 3; Table S3: Concentrations of identified compounds in ^1^H NMR analysis of ten commercial *Eucalyptus* hydrolat products.
