# Supplementary material for: Scent Detection Threshold of Trained Dogs to Eucalyptus Hydrolat
Source: Animals (Basel). 2024 Apr 3;14(7):1083. doi: 10.3390/ani14071083 (PMC11010826; doi:10.3390/ani14071083)
Supplement: Supplementary file 1 [file animals-14-01083-s001.zip › Supplementary Figure S1.pdf]

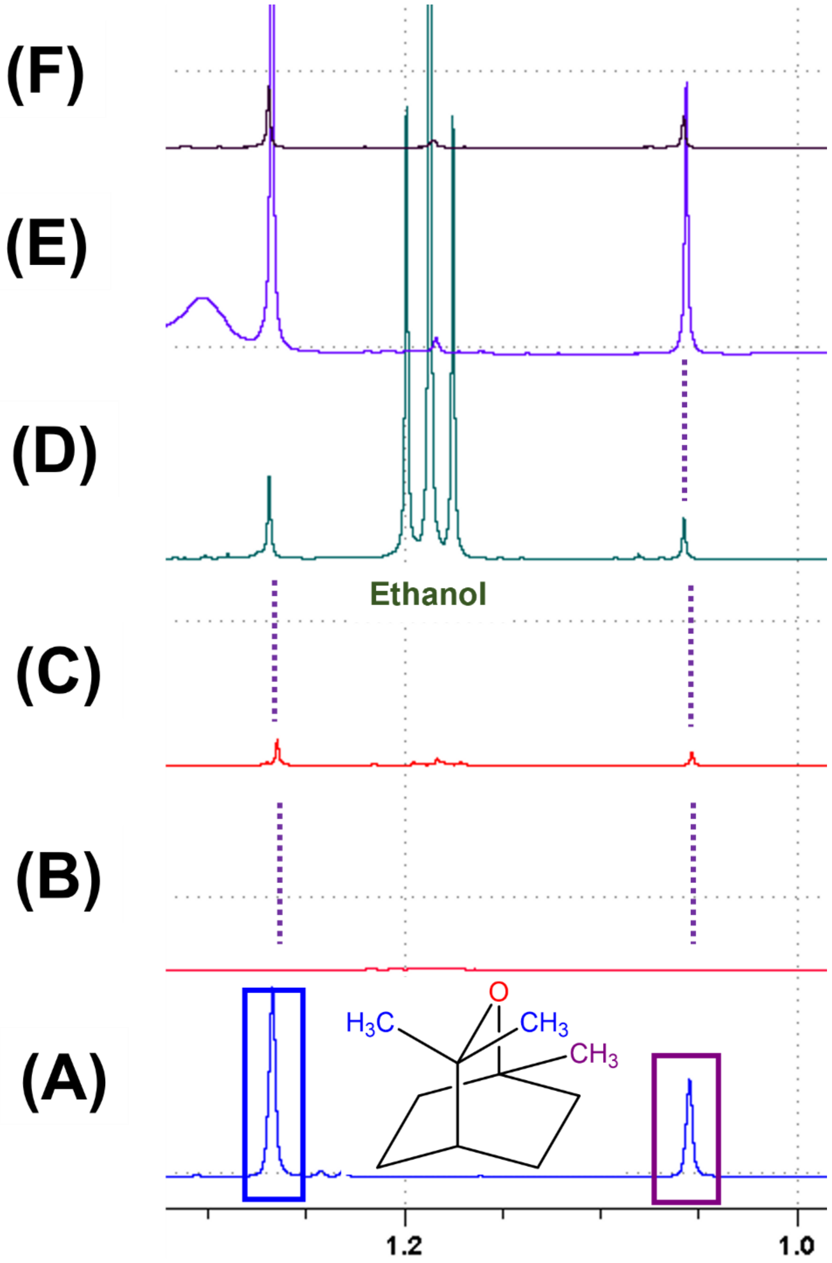

Figure S1. Expansion of methyl region of ^{1}H NMR spectra shows the structure of eucalyptol, the main compound in *Eucalyptus* oil. (A) Spectrum shows the assignment of the characteristic methyl signals (two blue and one purple CH_3 groups) of eucalyptol in the sample of 100% essential oil of *Eucalyptus radiata* (Frantsila, Kyröskoski, Finland). (B)-(F) Spectra show the corresponding signals in five different commercial *Eucalyptus* hydrolats. The purple dots illustrate the location of the signals. In addition, signals from ethanol are named in spectrum (D).
