# Supplementary material for: Scent Detection Threshold of Trained Dogs to Eucalyptus Hydrolat
Source: Animals (Basel). 2024 Apr 3;14(7):1083. doi: 10.3390/ani14071083 (PMC11010826; doi:10.3390/ani14071083)
Supplement: Supplementary file 1 [file animals-14-01083-s001.zip › Supplementary Tables S1-S3.pdf]

Table S1. Prepared dilutions in Study 2. Dilution ratios of *Eucalyptus* oil in water are depicted as the value of a volume fraction (mL:mL).

**solution**	**preparation of dilutions**	**dilution ratio**
stock solution	0.1 ml *Eucalyptus* oil, ad 1000 ml H_2O	$1:10^4$
Dilution 1	0.1 ml stock solution, ad 10 ml H_2O	$1:10^6$
Dilution 2	0.1 ml of dilution 1, ad 10 ml H_2O	$1:10^8$
Dilution 3	0.1 ml of dilution 2, ad 10 ml H_2O	$1:10^{10}$
Dilution 4	0.1 ml of dilution 3, ad 10 ml H_2O	$1:10^{12}$
Dilution 5	0.1 ml of dilution 4, ad 10 ml H_2O	$1:10^{14}$
Dilution 6	0.1 ml of dilution 5, ad 10 ml H_2O	$1:10^{16}$
Dilution 7	0.1 ml of dilution 6, ad 10 ml H_2O	$1:10^{18}$
Dilution 8	0.1 ml of dilution 7, ad 10 ml H_2O	$1:10^{20}$
Dilution 9	1.0 ml of dilution 8, ad 10 ml H_2O	$1:10^{21}$
Dilution 10	1.0 ml of dilution 9, ad 10 ml H_2O	$1:10^{22}$
Dilution 11	1.0 ml of dilution 10, ad 10 ml H_2O	$1:10^{23}$
Dilution 12	1.0 ml of dilution 11, ad 10 ml H_2O	$1:10^{24}$

The line highlights the change in preparation of dilution.

Table S2. Prepared dilutions in Study 3. Dilution ratios of *Eucalyptus* oil in water are depicted as the value of a volume fraction (mL:mL).

**solution**	**preparation of dilutions**	**dilution ratio**
stock solution	0.01 ml Eucalyptus oil, ad 1000 ml H_2O	$1:10^5$
Dilution 1	0.1 ml stock solution, ad 10 ml H_2O	$1:10^7$
Dilution 2	0.1 ml of dilution 1, ad 10 ml H_2O	$1:10^9$
Dilution 3	0.1 ml of dilution 2, ad 10 ml H_2O	$1:10^{11}$
Dilution 4	0.1 ml of dilution 3, ad 10 ml H_2O	$1:10^{13}$
Dilution 5	0.1 ml of dilution 4, ad 10 ml H_2O	$1:10^{15}$
Dilution 6	0.1 ml of dilution 5, ad 10 ml H_2O	$1:10^{17}$
Dilution 7	0.1 ml of dilution 6, ad 10 ml H_2O	$1:10^{19}$
Dilution 8	0.1 ml of dilution 7, ad 10 ml H_2O	$1:10^{21}$
Dilution 9	1.0 ml of dilution 8, ad 10 ml H2O	$1:10^{22}$
Dilution 10	1.0 ml of dilution 9, ad 10 ml H2O	$1:10^{23}$

The line highlights the change in preparation of dilution.

Table S3. Concentrations of identified compounds in ^{1}H NMR analysis of ten commercial *Eucalyptus* hydrolat products. The product names of hydrolats are coded as B-K.

Hydrolat	Eucalyptol	Bentzyl alcohol	Ethanol	Methanol	Polyethylene glycol (PEG)	Formic acid	Acetic acid
B	<0.01	33.69	<0.01	0.85	<0.01	0.46	0.93
C	0.26	41.54	0.25	0.61	<0.01	0.35	0.66
D	0.77	<0.01	27.63	0.98	<0.01	<0.01	0.06
E	5.05	<0.01	<0.01	<0.01	165.20	<0.01	<0.01
F	0.62	<0.01	<0.01	1.15	<0.01	<0.01	<0.01
G	0.10	41.32	0.12	0.93	<0.01	0.39	0.95
H	0.97	42.68	0.23	0.61	<0.01	0.35	0.64
I	<0.01	<0.01	16.77	0.95	<0.01	<0.01	0.85
J	0.41	<0.01	32.72	0.06	<0.01	<0.01	<0.01
K	<0.01	41.09	0.25	0.90	<0.01	0.39	0.76

The values are shown as mmol/L.
